# Retinal Remodeling: Concerns, Emerging Remedies and Future Prospects

**DOI:** 10.3389/fncel.2016.00038

**Published:** 2016-02-17

**Authors:** Vidhyasankar Krishnamoorthy, Pitchaiah Cherukuri, Deepak Poria, Manvi Goel, Sushma Dagar, Narender K. Dhingra

**Affiliations:** ^1^Department of Ophthalmology, University Medical Center GöttingenGöttingen, Germany; ^2^Developmental Neurobiology Laboratory, European Neuroscience Institute GöttingenGöttingen, Germany; ^3^National Brain Research CentreManesar, Haryana, India; ^4^Institute of Neuro- and Sensory Physiology, Heinrich-Heine UniversityDüsseldorf, Germany

**Keywords:** retinal degeneration, oscillatory activity, retinal prostheses, stem cells, optogenetics

## Abstract

Deafferentation results not only in sensory loss, but also in a variety of alterations in the postsynaptic circuitry. These alterations may have detrimental impact on potential treatment strategies. Progressive loss of photoreceptors in retinal degenerative diseases, such as retinitis pigmentosa and age-related macular degeneration, leads to several changes in the remnant retinal circuitry. Müller glial cells undergo hypertrophy and form a glial seal. The second- and third-order retinal neurons undergo morphological, biochemical and physiological alterations. A result of these alterations is that retinal ganglion cells (RGCs), the output neurons of the retina, become hyperactive and exhibit spontaneous, oscillatory bursts of spikes. This aberrant electrical activity degrades the signal-to-noise ratio in RGC responses, and thus the quality of information they transmit to the brain. These changes in the remnant retina, collectively termed “retinal remodeling”, pose challenges for genetic, cellular and bionic approaches to restore vision. It is therefore crucial to understand the nature of retinal remodeling, how it affects the ability of remnant retina to respond to novel therapeutic strategies, and how to ameliorate its effects. In this article, we discuss these topics, and suggest that the pathological state of the retinal output following photoreceptor loss is reversible, and therefore, amenable to restorative strategies.

## Introduction

**Figure 1:**
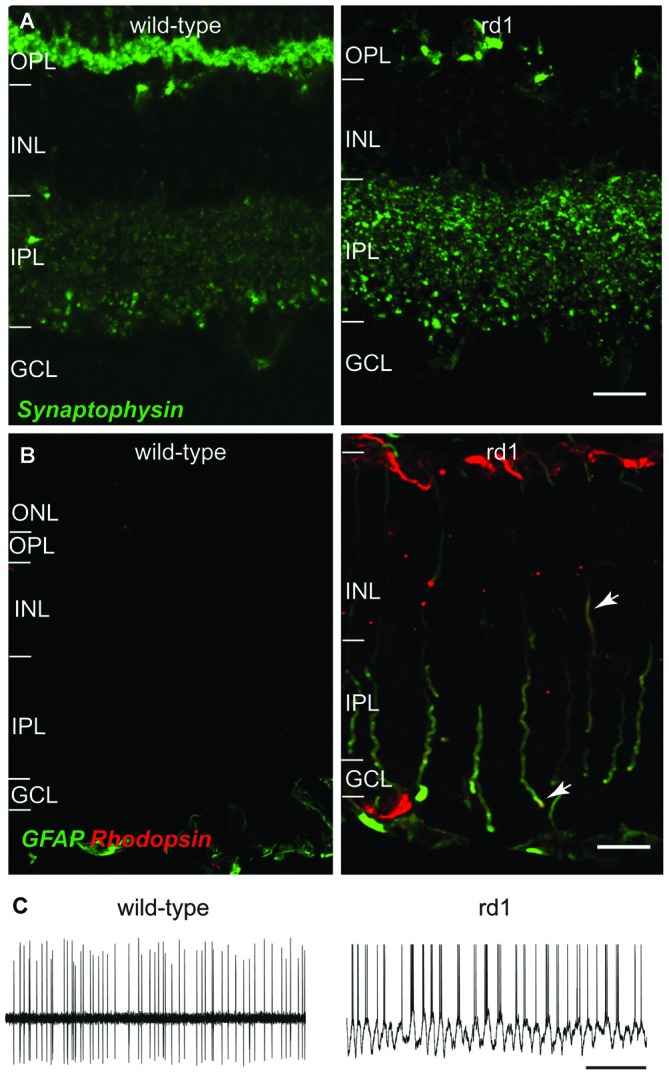
**Representative changes in retina following photoreceptor loss in rd1 mouse retina. (A)** Loss of photoreceptors results in upregulation of synaptic proteins in inner retina. Vertical retinal sections of adult wild-type (*left*) and rd1 (*right*) mouse retinas showing synaptophysin expression in OPL and IPL. Synaptophysin is nearly absent in the OPL, because the photoreceptor terminals have degenerated; however, synaptophysin expression is increased in the IPL. Scale bar: 50 μm. **(B)** Loss of photoreceptors in rd1 mouse causes several changes in Müller glial cells, including expression of several mature neuronal proteins. Vertical sections of wild-type (*left*) and rd1 (*right*) mouse retinas showing the expression of rhodopsin (*red*) in the GFAP-positive (*green*) Müller glia of rd1 mouse (*arrows* point to Müller cell processes expressing both GFAP and rhodopsin [*yellow*]). Scale bar: 10 μm. **(C)** The changes in inner retinal circuitry lead to oscillatory activity in RGCs. Representative spontaneous spike trains from RGCs in wild-type (*left*; extracellular recording) and rd1 (*right*; whole cell recording) mouse retinas showing oscillatory spiking in the rd1 mouse retina. Scale bar: 1 s. Images in **(A,B)** are adapted with permission from Dagar et al. (2014) and Goel and Dhingra (2012), respectively.

Loss of photoreceptors, as in retinitis pigmentosa and age-related macular degeneration, leads to extensive, phased, and regressive remodeling in inner retina (Strettoi and Pignatelli, 2000; Marc et al., 2003; Cuenca et al., 2005; Gargini et al., 2007; Barhoum et al., 2008; Nagar et al., 2009). The bipolar cells and horizontal cells show dendritic retraction, axonal sprouting, and ectopic synapse formation (Strettoi et al., 2002, 2003; Nagar et al., 2009). Some of the retinal neurotransmitter receptors, such as type-6 metabotropic glutamate receptors (mGluR6) and GABA_C_ receptors are downregulated, whereas others, such as AMPA, GABA_A_, and glycine receptors are upregulated following photoreceptor loss (Varela et al., 2003; Marc et al., 2007; Chua et al., 2009; Puthussery et al., 2009; Srivastava et al., 2015). The synaptic proteins in bipolar cells and amacrine cells (ACs) are upregulated, suggesting increased synaptic activity in these cell (Margolis et al., 2008; Borowska et al., 2011; Margolis and Detwiler, 2011; Dagar et al., 2014; Figure 1A). The ACs and retinal ganglion cells (RGCs) retain their gross morphology and receptive field properties, but they show significant molecular and physiological changes. These changes are accompanied by extensive changes in Müller cells (Figure 1B) that lead to formation of a glial seal (Strettoi et al., 2003; Nagar et al., 2009).

A significant outcome of photoreceptor degeneration is that the neuronal networks in inner retina start to oscillate spontaneously, resulting in oscillatory bursts of spikes in RGCs (Pu et al., 2006; Ye and Goo, 2007; Stasheff, 2008; Figure 1C). This degrades the signal-to-noise ratio in RGC responses, and thus the quality of information they transmit (Yee et al., 2012; Toychiev et al., 2013). The oscillatory activity has also been observed in the visual cortex of mice with retinal degeneration and may explain the mechanism underlying photopsia, the perception of spontaneous light flashes in blind patients (Dräger and Hubel, 1978; Heckenlively et al., 1988; Murtha and Stasheff, 2003; Bittner et al., 2009).

It is plausible that retinal remodeling sensitizes the degenerating retina to respond to novel therapeutic strategies, but it may also be a potential hurdle. It is unclear how therapeutic interventions, such as retinal prosthesis, cell/tissue transplantation, and gene therapy, interact with the molecular and circuitry-level changes in inner retina. Further, the glial seal presents a potential physical barrier for prosthetic microelectrodes and transplanted cells. Here, we present an overview of retinal remodeling and some of the emerging therapeutic strategies for retinal degeneration. Since at least some of the early retinal changes following photoreceptor loss are reversible, the future of treatment for retinal degeneration appears promising.

## Changes in Retinal Neurons

Loss of photoreceptors triggers a multitude of changes in second-order retinal neurons: bipolar cells and horizontal cells. Bipolar cells retract their dendrites following photoreceptor loss (Strettoi and Pignatelli, 2000; Strettoi et al., 2002; Nagar et al., 2009); however, in models where the photoreceptor loss is incomplete, the bipolar cells tend to form ectopic synapses with the surviving photoreceptors (Peng et al., 2000, 2003; Haverkamp et al., 2006; Marc et al., 2007; Chua et al., 2009; Puthussery et al., 2009). Photoreceptor loss results in reduced mGluR6 expression and reduced ionic currents in ON bipolar cells (Strettoi and Pignatelli, 2000; Varela et al., 2003; Gargini et al., 2007; Puthussery et al., 2009). Further, the ON cone bipolar cells aberrantly express functional ionotropic glutamate receptors (iGluRs; Marc et al., 2007; Chua et al., 2009). However, rod bipolar cells exhibit enhanced sensitivity to GABA (Varela et al., 2003). Horizontal cells decrease in number and show dendritic retraction and axonal growth into the inner nuclear layer (INL) and inner plexiform layer (Strettoi and Pignatelli, 2000; Park et al., 2001; Strettoi et al., 2003; Nagar et al., 2009).

The third-order retinal neurons, ACs and RGCs, have been shown to generally maintain their dendritic geometry, stratification pattern, and intrinsic and receptive field properties (Strettoi and Pignatelli, 2000; Strettoi et al., 2003; Margolis et al., 2008; Mazzoni et al., 2008; Lin and Peng, 2013). However, in late stages, RGCs show altered dendritic branching patterns and even migrate to INL (Marc and Jones, 2003; Jones and Marc, 2005; Jones et al., 2005; O’Brien et al., 2014). In fact, the ACs have been shown to undergo significant biochemical and morphological changes early on. For example, AC-specific synaptic proteins, synapsin-I and syntaxin-I are upregulated (Dagar et al., 2014); calbindin-positve ACs progressively loose functional NMDA receptors (Chua et al., 2009); AII ACs show reduced dab-1 expression and progressively smaller lobular appendages (Barhoum et al., 2008); and putative AII ACs (that express GlyT-1 transporter) extend their processes into and even migrate to the OPL (Park et al., 2004).

## Changes in MÜller Glial Cells

Müller glia are involved in several retinal functions, including photoreceptor metabolism, pH maintenance, blood-retina barrier formation, neurotransmitter reuptake, and photopigment recycling (Bunt-Milam and Saari, 1983; Das et al., 1992; Tout et al., 1993; Newman and Reichenbach, 1996). Following photoreceptor degeneration, Müller cells undergo hypertrophy and reactive gliosis, characterized by upregulation of glial fibrillary acidic protein, eventually leading to formation of a glial seal (Strettoi et al., 2002; Nagar et al., 2009; Goel and Dhingra, 2012). The glial seal isolates the remnant neural retina from the retinal pigment epithelium and choroid and could potentially act as a physical barrier for bionic and cell-based strategies aimed at restoring vision (Henriksen et al., 2014). Müller cells have also been shown to dedifferentiate and re-enter cell cycle following photoreceptor loss (see below).

## Spontaneous Oscillatory Activity in inner Retina

The retinal remodeling results in dramatic physiological changes in retinal output. Specifically, RGCs start to produce spontaneous oscillatory bursts of spikes and exhibit reduced signal-to-noise ratio in their light responses (Pu et al., 2006; Margolis et al., 2008; Stasheff, 2008; Ryu et al., 2010a,b; Yee et al., 2012; Toychiev et al., 2013).

The oscillatory activity does not originate in RGCs, but is presynaptic (Margolis et al., 2008; Borowska et al., 2011; Menzler and Zeck, 2011). ON-cone bipolar cells, AII ACs, and the gap junctions connecting them may be the source loci (Borowska et al., 2011; Menzler and Zeck, 2011; Trenholm et al., 2012; Choi et al., 2014; Margolis et al., 2014). Both bipolar cells and ACs have been shown to exhibit regenerative activity in *in vitro* preparations (Solessio et al., 2002; Ma and Pan, 2003; Cembrowski et al., 2012). Interestingly, even remnant cones and horizontal cells exhibit spontaneous oscillatory activity after photoreceptor loss (Haq et al., 2014). The oscillatory activity was recently shown to originate in ON pathway and transfer to OFF pathway via glycinergic, likely the AII ACs (Poria and Dhingra, 2015). As a result, the oscillatory activities in ON and OFF RGCs are 180° out of phase (Margolis et al., 2014). Overall, these observations show that the inner retinal neurons start to oscillate following photoreceptor loss. However, it is not clear how this oscillatory activity would interact with therapeutic interventions, such as retinal prostheses.

## Approaches to Treat Retinal Degeneration

### Preventive Strategies

Attempts to prevent or slow down the progression of photoreceptor degeneration have shown promise. For example, inhibiting ceramide biosynthesis pathway with myriocin or activating Wnt/β-catenin signaling in the Müller cells slow down the disease progression in rd10 mouse (Strettoi et al., 2010; Patel et al., 2015). Interestingly, raising rd10 mice in enriched environment has also been shown to delay the progression of photoreceptor loss, possibly due to increased expression of CNTF and mTOR (Barone et al., 2012, 2014). Slowing down of photoreceptor degeneration by enriched environment may provide additional benefit if combined with other treatment approaches to restore visual function, not only during development but also in adulthood.

### Cell Transplantation

There have been significant attempts to transplant cells of various types in degenerating retina, with varied results. Early studies showed the survival and integration of stem cells, such as embryonic or hematopoietic stem cells in rd mouse retina (Otani et al., 2002, 2004; Meyer et al., 2006). The transplanted photoreceptor precursors have been shown to form rudimentary synapses with rod bipolar cells and partially restore visual function (MacLaren et al., 2006). Recently, Gonzalez-Cordero et al. (2013) transplanted mouse embryonic stem cell-derived photoreceptor precursors in Gnat^−/−^ mouse model, and found that they integrated into the host retina and differentiated to form synapse-like structures. Human embryonic stem cells and induced pluripotent stem cells have also shown promise in restoring vision (Reynolds and Lamba, 2014; Wright et al., 2014).

The glial seal formed by Müller cells is a concern for transplantation strategies, because it can potentially limit the migration of transplanted cells into the retinal layers (Kinouchi et al., 2003). However, pharmacological tools, such as alpha-amino adipic acid or chondroitinase ABC that break the glial seal, could facilitate the migration and integration of the transplanted cells (West et al., 2008; Barber et al., 2013). Alpha-amino adipic acid is a glial toxin that, depending on the dose, can exert a variety of effects on Müller cells, from cell proliferation to cell death and therefore requires careful dose titration to facilitate transplantation (Olney, 1982; Takeda et al., 2008; West et al., 2008). Another concern is related to the ability of the second-order retinal neurons in degenerating retina to receive synaptic inputs from the transplanted cells. Enabling bipolar cells and horizontal cells to extend their dendrites in the absence of photoreceptors would require understanding the signaling pathways that regulate dendritic growth and lamination in these neurons. This is particularly challenging, because such signaling pathways are likely absent or dormant in adult mammalian retina (D’Orazi et al., 2014).

### Gene Therapy and Optogenetic Approaches

Gene therapy offers a promising approach to restore vision. Several studies have shown that introducing a missing gene can lead to partial or complete restoration of vision in animal models (Ali et al., 2000; Acland et al., 2001; Alexander et al., 2007; Mancuso et al., 2009; Michalakis et al., 2010; Beltran et al., 2012). The success in animal models has led to human clinical trials to treat Leber’s congenital amaurosis (Bainbridge et al., 2008; Cideciyan et al., 2008; Hauswirth et al., 2008; Maguire et al., 2008). However, although this approach showed improvement in visual function in the short term (~1–3 years), the retina continued to degenerate and visual sensitivity deteriorated over the longer term (~3–6 years; Cideciyan et al., 2013; Bainbridge et al., 2015; Jacobson et al., 2015; Wright, 2015). Designing more efficient vectors for gene delivery and combining gene therapy with measures that slow or arrest retinal degeneration may help prolong the therapeutic efficacy (Cideciyan et al., 2013; Wright, 2015). Another challenge that the gene therapy faces is that inherited retinal degeneration is a heterogeneous disease involving diverse genes, which requires developing mutation-specific therapy for each disease subtype (Hartong et al., 2006; Dalkara et al., 2015).

Alternative approaches that impart light sensitivity by introducing opsins into specific remnant retinal neurons have shown tremendous potential. For example, introducing melanopsin, a native light sensor normally present in a small subset of RGCs, into a wider population of RGCs in mouse resulted in a partial rescue of vision (Lin et al., 2008). However, melanopsin is a high-threshold opsin, which limits its clinical use. Recently, expressing a chimeric protein (opto-mGluR6) that combined mGluR6 and melanopsin in ON bipolar cells produced simple visual behaviors in rd1 mouse (van Wyk et al., 2015). Lagali et al. (2008) expressed channel-rhodopsin-2 (ChR2), a microbial light-gated channel that depolarizes the cell on light exposure, in ON bipolar cells and showed light responses in RGCs; these mice were able to perform optomotor tasks. Similarly, Zhang et al. (2009) expressed the depolarizing ChR2 and hyperpolarizing halorhodopsin in RGCs and showed ON and OFF light responses. In an elegant set of experiments, Busskamp et al. (2010) expressed ChR2 and halorhodopsin in the remnant cone photoreceptors and were able to partially restore vision in blind mice.

Overall, these data demonstrate the tremendous potential of genetic and optogenetics-based approaches in restoring vision. Targeting specific channels in specific cells may be required to achieve more clinically-relevant outcomes. Similarly, a deeper understanding of the changes in gene expression patterns and genetic markers in the remnant retinal neurons would help design specific targeting vectors to successfully treat retinal degeneration.

### Photoswitches

Photoswitches are molecules that reversibly change their conformation in response to specific wavelengths of light. Acrylamide-azobenzene-quaternary ammonium (AAQ) and a modified AAQ, DENAQ, act as photoswitches for the K^+^ channels: their *trans* form increases neuronal excitability, whereas the *cis* form decreases neuronal excitability. A single intra-ocular injection of a photoswitch has been shown to confer light sensitivity to RGCs (Polosukhina et al., 2012; Tochitsky et al., 2014). However, these molecules are not cell specific. This could be overcome by engineering a photoswitch that can be targeted to specific cells. A photoswitch expressed with light-gated ionotropic glutamate receptor (LiGluR) specifically in RGCs or ON bipolar cells has been shown to restore vision in mice and canine models of retinal degeneration (Caporale et al., 2011; Gaub et al., 2014).

### Retinal Prostheses

Producing artificial vision in blind animals and humans by electrically stimulating the degenerating retina with a prosthetic “chip” is no longer a fantasy. There are two approaches to stimulating the retina: subretinal, where a light-sensitive photodiode array substitutes the missing photoreceptors and stimulates the remnant circuit, and epiretinal, where RGCs are stimulated directly. Devices of both types have been approved for human use and provide measurable visual acuity and rudimentary object localization/recognition (Ahuja et al., 2011; Zrenner et al., 2011; Humayun et al., 2012; da Cruz et al., 2013; Kotecha et al., 2014; Ho et al., 2015; Stingl et al., 2015).

Considering that inner retina responds remarkably to loss of photoreceptors, a biological process, it is unclear how it would respond to an artificial device in the long term. Although the initial success is promising, more work is required to improve specificity, spatial and temporal resolution, contrast sensitivity, and intraocular packaging (Eiber et al., 2013; Zrenner, 2013; Maghami et al., 2014; Weiland and Humayun, 2014). For example, targeting specific ganglion cells could help mimic natural aspects of visual processing (Dorn et al., 2013). Recent work in isolated primate retina showed the potential of improved prosthetic designs in eliciting precisely timed spikes to produce spatiotemporal patterns in RGCs similar to those produced by light stimuli (Jepson et al., 2014a,b). Recent advances in powering a subretinal electrode array wirelessly will resolve the problem of implanting the cumbersome battery packs (Mathieson et al., 2012; Mandel et al., 2013; Lorach et al., 2015a,b). In a different approach, optoelectronic polymer interface have been shown to impart light sensitivity to degenerate retina *ex-vivo*, both in subretinal and epiretinal configurations (Ghezzi et al., 2013; Gautam et al., 2014). Even with these significant advances, it remains unclear how a subretinal prosthetic device would employ the remodeled remnant retinal circuitry or how an epiretinal device would stimulate specific RGCs through the glial seal to produce meaningful vision for blind patients.

### Endogenous Regeneration

Several recent discoveries suggest that the endogenous regenerative capacity of retina can be exploited for repair. Specifically, Müller glia have been shown to exhibit stem cell properties. In response to retinal injury in fish, Müller cells and the ciliary marginal zone cells can regenerate all retinal neurons, resulting in complete functional recovery (Braisted et al., 1994; Fimbel et al., 2007; Thummel et al., 2008). Since Müller glia are among the last retinal cells to develop, it is possible that they do not undergo the irreversible cell fate determination event (Young, 1985; Cepko et al., 1996; Jadhav et al., 2009). They share 43% similarity in the expressed genes with the retinal progenitor cells and retain most of the genes expressed by the late retinal progenitor cells (Blackshaw et al., 2004; Livesey et al., 2004; Roesch et al., 2008; Jadhav et al., 2009). Müller glia have been considered the dormant stem cells of retina (Jadhav et al., 2009).

The mammalian Müller glia, however, have limited regenerative capacity. They have been shown to re-enter cell cycle and express stem-cell and mature-retinal-cell markers, such as PKC-α, NSE, recoverin, rhodopsin, calretinin, NeuN, and prox1 following photoreceptor loss, but they do not generate functional photoreceptors (Ooto et al., 2004; Karl et al., 2008; Wan et al., 2008; Zhao et al., 2010; Goel and Dhingra, 2012; Greferath et al., 2015).

Several approaches to promote neuroprotective and regenerative capacity of Müller glia in mammalian retina seem promising. Müller glia secrete several neuroprotective growth factors, such as bFGF, BDNF, GDNF, NGF, and CNTF (Wen et al., 1995; Chu et al., 1998; Liu et al., 1998; Harada et al., 2000; Peterson et al., 2000; Delyfer et al., 2005; Hauck et al., 2006). Application of FGF and CNTF helps mammalian Müller glia differentiate into retinal neurons *in vitro* (Lawrence et al., 2007; Giannelli et al., 2011; Jayaram et al., 2014). Müller glia could be used as scaffolds to express neuroprotective molecules, such as GDNF, to slow down the progression of degeneration (Klimczak et al., 2009; Dalkara et al., 2011). Expressing specific genes in Müller glia can lead to their differentiation into specific retinal neurons (Ooto et al., 2004). In addition, transcriptomic analyses of Müller glia from degenerating retinas have revealed changes in glutathione metabolism and peroxide detoxification, which could potentially confer neuroprotection (Roesch et al., 2012).

## Conclusions and Future Directions

More than a decade of research has revealed the molecular to circuit-level details of retinal remodeling, but several challenges remain. For example, it is unclear how events, such as dendritic retraction, aberrant neuritogenesis, cell migration, and ectopic synapse formation, are connected temporally. We know how some retinal neurotransmitter receptors or ion channels respond to loss of photoreceptors, but it is unclear how this occurs at the level of specific cell types.

Longitudinal transcriptional profiling of specific cells may help identify potential signaling pathways and molecular targets, as has been done for amyotrophic lateral sclerosis (Saxena et al., 2009). The GENSAT mouse lines expressing GFP in specific retinal cells may also be valuable (Siegert et al., 2009, 2012). Specific cells can be sorted by FACS, and their transcriptome analyzed by RNA sequencing during or following photoreceptor degeneration; this could help identify the signaling molecules and pathways involved in remodeling, and thus potentially help discover novel interventional strategies (Sharma et al., 2015; Yang et al., 2015).

Many of the promising treatment approaches, such as retinal prostheses, gene therapy, optogenetics, photoswitches, and endogenous regeneration will likely benefit from advances in specific cell targeting. One possibility that has not been explored sufficiently is to combine these treatment approaches. A recent study combined optogenetic expression of ChR2 in RGCs with retinal prostheses and showed that driving the stimulator with retina’s neural code elicits RGC firing similar to normal retina (Nirenberg and Pandarinath, 2012). It is plausible that combining prosthetic stimulation or gene therapy with approaches, such as pharmacological agents that slow down the degeneration or promote endogenous regeneration, or those that abolish oscillatory activity in degenerated retina could expedite the progress in restoring vision for blind patients.

Recent work on biochemical mechanisms underlying oscillatory activity in retina may help design newer drug therapies for retinal degeneration. For example, blocking gap junctions with meclofenamic acid has been shown to eliminate oscillatory activity in RGCs (Trenholm et al., 2012; Menzler et al., 2014). Similarly, blocking glycinergic signaling with strychnine removes oscillatory activity in OFF RGCs (Poria and Dhingra, 2015). Although more work is required, a notable outcome is that oscillatory activity in RGCs is acutely reversible, implying that it is not a result of any massive or structural remodeling and that the biochemical and physiological changes in inner retina may be amenable to restoration strategies (Dagar et al., 2014).

## Author Contributions

All listed authors, have made substantial, direct, and intellectual contribution to the work, and approved it for publication.

## Funding

This study was funded by National Brain Research Centre, India.

## Conflict of Interest Statement

The authors declare that the research was conducted in the absence of any commercial or financial relationships that could be construed as a potential conflict of interest.
